# Unsupervised Learning of Overlapping Image Components Using Divisive Input Modulation

**DOI:** 10.1155/2009/381457

**Published:** 2009-05-05

**Authors:** M. W. Spratling, K. De Meyer, R. Kompass

**Affiliations:** ^1^Division of Engineering, King's College London, London WC2R 2LS, UK; ^2^Centre for Brain and Cognitive Development, Birkbeck College, University of London, London WC1E 7HX, UK; ^3^Artificial Intelligence Group, Institute of Computer Science, Freie Universität Berlin, 14195 Berlin, Germany

## Abstract

This paper demonstrates that nonnegative matrix factorisation is mathematically related to a class of neural networks that employ negative feedback as a mechanism of competition. This observation inspires a novel learning algorithm which we call Divisive Input Modulation (DIM). The proposed algorithm provides a mathematically simple and computationally efficient method for the unsupervised learning of image components, even in conditions where these elementary features overlap considerably. To test the proposed algorithm, a novel artificial task is introduced which is similar to the frequently-used bars problem but employs squares rather than bars to increase the degree of overlap between components. Using this task, we investigate how the proposed method performs on the parsing of artificial images composed of overlapping features, given the correct representation of the individual components; and secondly, we investigate how well it can learn the elementary components from artificial training images. We compare the performance of the proposed algorithm with its predecessors including variations on these algorithms that have produced state-of-the-art performance on the bars problem. The proposed algorithm is more successful than its predecessors in dealing with overlap and occlusion in the artificial task that has been used to assess performance.

## 1. Introduction

Images are often composed of a relatively small set of elementary features or components. These components may overlap with, or partially occlude, other components in a visual scene. Vision systems attempting to recognise objects as a combination of such elementary features need to be capable of two things. Firstly, parsing complex images into elementary components, even if these are partially occluded due to overlap. Secondly, extracting a meaningful and relatively sparse representation set (i.e., learning the elementary components) from cluttered and complex images. In this paper we present a novel neural network algorithm that is capable of both accurately parsing images into elementary components and reliably learning image components, even when these components are heavily overlapping. The proposed algorithm is mathematically simple, computationally efficient, and biologically plausible.

Nonnegative matrix factorisation (NMF) is an existing method that has been specifically proposed for the unsupervised learning of image components [1–7]. Without additional constraints on the factorisation, NMF fails to deal successfully with occlusion even in simple, artificial, tasks where overlap occurs [8]. It has been suggested previously [9] that NMF can be interpreted as a divisory form of feedback inhibition, as used in the preintegration lateral inhibition/dendritic inhibition model [10–12] and negative feedback networks [13–18]. This mathematical similarity between NMF and negative feedback networks will be described fully in Section 2. One difference between these algorithms is that NMF operates in batch mode, while negative feedback networks are online learning algorithms. In Section 2.2.1 we develop an online implementation of NMF which serves to explicitly illustrate the analogy between NMF and negative feedback networks. However, this new implementation of NMF suffers from the same deficiencies as the original NMF algorithm when dealing with overlap between input components. We therefore propose modifications to the sequential version of NMF to overcome these problems (Section 2.3). The resulting algorithm shares features of both NMF and negative feedback networks. It can also be interpreted as a neural implementation of Bayesian inference (Section 2.4). In comparisons of the proposed algorithm with its predecessors we show that it has significantly better performance in both parsing (Section 3.2) and learning (Section 3.3) overlapping image components for a novel artificial task (the “squares problem.”) Furthermore, Section 3.4 demonstrates that the proposed algorithm outperforms, or has equal performance to, a number of variations on these previous algorithms including ones that have produced state-of-the-art performance on a similar artificial task (the “bars problem.”) Results are also provided (in Section 3.5) for learning components from real image data.

## 2. Methods

**Figure 1:**
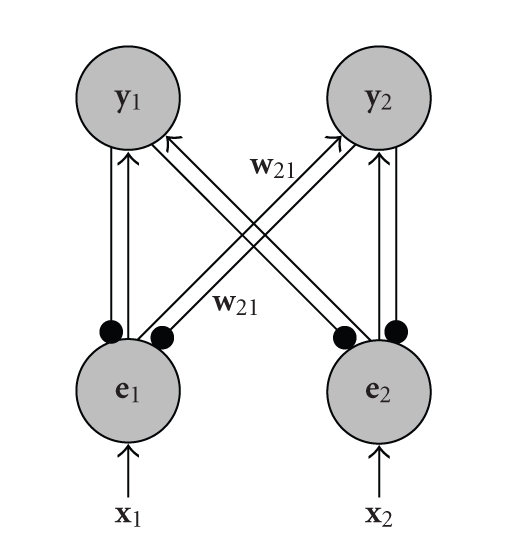
A simple two-node, two-input, neural network illustrating the architecture employed by all the algorithms described in this article. Nodes are shown as large circles, excitatory synapses as arrows and inhibitory synapses as small filled circles. Reciprocal feedforward and feedback connections have identical strengths.

All of the algorithms described below can be interpreted as neural networks with an architecture like that illustrated in Figure 1. This architecture can be understood both as a generative model (one in which the output activation produces, via a set of feedback connections, a reconstruction of the input stimulus) or as a recognition model (one in which the inputs are mapped, via a set of feedforward weights, onto a pattern of neural activations which “represent” the stimulus) [19]. From both these perspectives a vector **x** denotes the inputs to the network, and **y** represents the outputs of the network. However, the meaning given to the values of **e** varies. In generative terms, each element of **e** represents the residual error between an input and the reconstruction of the input generated, via the feedback connections, from the outputs of the network. In recognition terms, these feedback connections can be interpreted as providing a form of lateral inhibition that targets the inputs to a population of competing nodes, each element of **e** thus represents the corresponding input value following inhibition. These different perspectives do not specify changes to the underlying mathematical model. Rather, the same model can simply be interpreted in different ways. We will therefore use the terms “reconstruction error” and “inhibited inputs” and the terms “feedback” and “lateral” interchangeably.

### 2.1. Negative Feedback Networks

Competition between nodes in a neural network is an essential feature of many unsupervised learning algorithms. It is used to make the synaptic weights of individual nodes more distinct, and hence to enable nodes to be selective for different input stimuli. Lateral inhibition, in which nodes inhibit the outputs of other nodes, is one mechanisms that is commonly used to provide competition in unsupervised neural network algorithms (see [12], for references). However, an alternative mechanism is to use inhibition that targets the inputs to a population of competing nodes. In such a network [10–18] activation is fed back from the output nodes to subtractively inhibit the inputs to those nodes, as illustrated in Figure 1. Two different algorithms of this type are described in what follows.

#### 2.1.1. Fyfe's Negative Feedback Network

In the negative feedback network algorithm proposed by Fyfe and his colleagues [13, 14, 16], for a network with *m* inputs and *n* nodes, the network activity is calculated as

(1) y=Wx,

(2) $$e=x-W^{T}y,$$

where **y** = [*y*_1_,…, *y*_*n*_]^*T*^ is a vector of output activations, **x** = [*x*_1_,…, *x*_*m*_]^*T*^ is a vector of input activations, **W** = [**w**_1_,…, **w**_*n*_]^*T*^ is an *n* by *m* matrix of weight values, each row of which contains the weights received by a single node, and **e** is the inhibited value of the input (or, equivalently, the reconstruction error). For each new input pattern, the values of **y** and **e** are calculated without iteration. Therefore, inhibition has no effect on the response of the network, and is only used to affect the synaptic weights via the following learning rule:

(3) $$W⟵W+\beta ye^{T},$$

where *β* is a parameter controlling the learning rate. In order to learn the elementary components of images it is necessary to prevent the occurrence of negative values, by clipping negative weights at zero, that is, by setting *w*_*ji*_ = 0 if *w*_*ji*_ < 0 [13, 14, 16].

There is no competition in this architecture. Inhibition only serves to affect learning (and hence the selectivities of the nodes in the long term), but does not affect the output of the nodes in the short term in response to the current stimulus. This lack of competition results in the network failing to correctly represent the input it receives even if nodes have correctly learnt weights that are selective to patterns within the stimulus. This problem will be illustrated in Section 3.2.

#### 2.1.2. Harpur's Negative Feedback Network

The negative feedback network proposed by Harpur et al. [17, 18, 20] does allow the competition to affect the output response of the network, and hence to affect the selectivities of the nodes in the short term. Network activity is determined using the following equations:

(4) $$e=x-W^{T}y,$$

(5) y⟵y+μWe.

For each new input image, the output values (**y**) are initialised to zero, and then the above equations are iterated to find the final values for **y** and **e**. At each iteration, negative values of **y** are clipped by making them equal to zero. The parameter *μ* is a scale factor controlling the rate at which the output activations change during this iterative process. It should be noted that if *μ* is too large this can cause certain values within **y** to become large in a single step. This will subsequently cause the elements in **e** which provide the inputs to the highly active nodes to become small, or negative, resulting in the corresponding node activations becoming small at the next step. The output activities will thus oscillate between high and low values and never reach a steady state. To avoid such instability it is necessary to use small positive values of *μ* which means that **y** is updated slowly and many iterations are required to allow convergence to the steady-state values.

The learning rule, proposed in Harpur and Prager [20], is identical to that used by Fyfe's algorithm:

(6) $$W⟵W+\beta ye^{T}.$$

Following learning, weights are clipped to be in the range [0, 1].

### 2.2. Nonnegative Matrix Factorisation

Nonnegative matrix factorisation is a method that seeks to find factors, **W** ^*T*^ and **Y**, of a nonnegative matrix **X** under the constraint that both factors contain only elements with nonnegative values, such that

(7) $$X\approx W^{T}\text{Y}.$$

It has been proposed that this method is particularly suitable for finding the parts-based decompositions of images [1–7], since from the physical properties of image formation it is known that image components are nonnegative and that these components are never subtracted in order to generate images. In this case **X** = [**x**_1_,…, **x**_*p*_] is an *m* by *p* matrix of training images each column of which contains the pixel values of an image, **W** ^*T*^ is an *m* by *n* matrix of weight values the columns of which represent components (or basis vectors) into which the images can be decomposed, and **Y** = [**y**_1_,…, **y**_*p*_] is an *n* by *p* matrix describing the activation of each component in the corresponding training image. An individual training image (**x**_*k*_) can therefore be reconstructed such that **x**_*k*_ ≈ **W** ^*T*^ **y**_*k*_.

Several different algorithms have been proposed for finding the factors **W** ^*T*^ and **Y** under nonnegativity constraints. One such algorithm [21] minimises the Kullback-Leibler divergence between the training images (**X**) and the reconstructed images (**W** ^*T*^ **Y**). In this algorithm, the update rules for the node activations and weights are

(8) $$\text{Y}⟵\text{Y}⊗\left(W\left\{X⊘\left(W^{T}\text{Y}\right)\right\}\right)⊘\overset{˜}{W},$$

(9) $$W^{T}⟵W^{T}⊗\left(\left\{X⊘\left(W^{T}\text{Y}\right)\right\}{\text{Y}}^{T}\right)⊘\overset{˜}{\text{Y}},$$

where $\overset{˜}{W}$ is an *n* by *p* matrix each column of which contains the sum of the weights corresponding to each basis vector (i.e., each column equals [∑_*i*=1_^*m*^ *w*_1*i*_,…,∑_*i*=1_^*m*^ *w*_*ni*_]^*T*^), $\overset{˜}{\text{Y}}$ is an *m* by *n* matrix each row of which is equal to the activation of each component summed over all the training images (i.e., each row equals [∑_*k* = 1_^*p*^ *y*_1*k*_,…,∑_*k* = 1_^*p*^ *y*_*nk*_]), and ⊘ and ⊗ indicate element-wise division and multiplication, respectively.

#### 2.2.1. Sequential NMF

In this section, we develop a sequential implementation of the NMF algorithm. This new implementation serves two purposes. Firstly, it helps to demonstrate the similarity between NMF and negative feedback networks. Secondly, it provides a link between NMF and the new algorithm we propose in Section 2.3.

In analogy with the term **e** used in the negative feedback networks, we introduce the term **E** = **X** ⊘ (**W** ^*T*^ **Y**). **E** is an *m* by *p* matrix the elements of which can be considered to represent the residual error between the input (**X**) and the top-down reconstruction of the input (**W** ^*T*^ **Y**), or equivalently, the inhibited input to a population of competing nodes. Substituting **E** into (8) and (9) and taking the transpose of each side of the equation for updating the weights, allows the NMF update rules to be rewritten as

(10) $$\begin{array}{c} \text{Y}⟵\text{Y}⊗\left(WE\right)⊘\overset{˜}{W},\\ W⟵W⊗\left(\text{Y}E^{T}\right)⊘{\overset{˜}{\text{Y}}}^{T}. \end{array}$$

In contrast to the negative feedback neural networks, NMF uses a batch, rather than a sequential, update procedure. Hence **Y**, **X**, and **E** are matrices of activations, input images, and reconstruction errors for all training data, rather than vectors for a single training image (i.e., **y**, **x**, and **e**) as used in the negative feedback networks. However, the output activations generated by NMF for any one training image are independent of the responses to other images. Hence, for a single training image the output activations are given by

(11) $$y⟵y⊗\left(We\right)⊘\overset{˜}{w},$$

where $\overset{˜}{w}$ is a single column of $\overset{˜}{W}$ and **e** is as defined in (12). Each element of vector $\overset{˜}{w}$ represents the sum of the weights forming each basis vector (or equivalently the total synaptic weight received by one output node).

(12) $$e=x⊘\left(W^{T}y\right).$$

An outstanding question is how are the values of **y** calculated? In the batch version of NMF, the activation values are randomly initialised before the first epoch of training and are subsequently updated each epoch using the values calculated in the previous update as the initial values for the next update. The final response of the network is therefore only generated after many epochs, when all the stimuli have been presented to the network multiple times. In a sequential algorithm, the response to the current stimulus is required immediately, and the response that was generated previously to the same stimulus is unknown. Hence, a sequential version of NMF will necessarily vary from the batch version in terms of how the values of **y** are calculated.

In order to generate a response to each stimulus as it is presented to the network the method employed by Harpur's negative feedback network can be used. For each new input pattern, the output values (**y**) need to be initialised, and then the above equations iterated to find the final values for **y** and **e**. Several methods suggest themselves for initialising **y** when each new image is presented. One option would be to randomly initialise the activations. However, this may result in many false parsings due to nodes that provide the correct representation being randomly assigned a small initial activation which prevents them from becoming strongly active. Another option would be to set $y=\left(Wx\right)⊘\overset{˜}{w}$. However, doing this directly is difficult to justify biologically, as it would require the output node activations to be calculated directly from the inputs, by-passing the error-detecting nodes, on each occasion when a new image was presented. However, the same result could be obtained by initialising the output activations to zero, and modifying the activation functions as follows:

(13) $$e=x⊘\left(\epsilon +W^{T}y\right),$$

(14) $$y⟵\left(\epsilon +y\right)⊗\left(We\right)⊘\overset{˜}{w}.$$

The parameter *ϵ* is a small constant (i.e., 1 × 10^−10^) that has a negligible effect on the calculation of **e** and **y** except when the values of **y** are approximately zero, or equivalently, when the input has been blank (i.e., *x*_*i*_ = 0 ∀ *i*) causing the output activations to become zero. If this is the case, then at the first iteration after a new stimulus is presented, the residual error becomes **e** = **x** ⊘ *ϵ* (from (13)). The output of the network is then calculated as $y=(\epsilon +0)⊗\left(W\left(x⊘\epsilon \right)\right)⊘\overset{˜}{w}=Wx⊘\overset{˜}{w}$ (from (14)). The parameter *ϵ* is also useful to prevent division-by-zero errors in the calculation of **e**. The stability of the original NMF algorithm is also improved by using a small constant to prevent division-by-zero errors in both (8) and (9). This modification is actually essential for the batch NMF algorithm to be applied successfully to the artificial task considered in Section 3.

The synaptic weight updates in NMF are a function of all the training images. However, we can derive an equivalent learning rule that can be applied to single training images presented in sequence. The weight update rule can be rewritten as

(15) $$W⟵W⊗\left({\overset{˜}{\text{Y}}}^{T}+\text{Y}\left(E^{T}-1\right)\right)⊘{\overset{˜}{\text{Y}}}^{T}.$$

Hence,

(16) $$\begin{array}{c} W⟵W⊗\left(1+\left(\text{Y}\left(E^{T}-1\right)⊘{\overset{˜}{\text{Y}}}^{T}\right)\right),\\ W⟵W⊗\left(1+\overset{p}{\underset{k=1}{\sum }}\left(y_{k}\left(e_{k}^{T}-1\right)⊘{\overset{˜}{\text{Y}}}^{T}\right)\right), \end{array}$$

where **y**_*k*_ and **e**_*k*_ are the node activations and reconstruction errors for a single training image *k*. If the weights are updated sequentially using the following rule:

(17) $$W_{k}=W_{k-1}⊗\left(1+y_{k-1}\left(e_{k-1}^{T}-1\right)⊘{\overset{˜}{\text{Y}}}^{T}\right),$$

where **W**_*k*_ denotes the weight values after training on the *k*th image. Then **W**_*k*_ → **W** as *k* → *p*, assuming that we can ignore all higher order terms of the form $y_{1}y_{2}(e_{1}^{T}-1)(e_{2}^{T}-1)⊘{{\overset{˜}{\text{Y}}}^{2}\;}^{T},\;\;y_{1}y_{2}y_{3}(e_{1}^{T}-1)(e_{2}^{T}-1)(e_{3}^{T}-1)⊘{{\overset{˜}{\text{Y}}}^{3}\;}^{T}$, and so forth. This is justified since the activation values will be fractional and the $\overset{˜}{\text{Y}}$ values are likely to be large.

In an online learning algorithm the values of $\overset{˜}{\text{Y}}$ (the activation of each node summed over all the training images) are unknown. It would be possible to estimate these values by averaging the output activations over a large number of training examples. However, for simplicity we replace $\overset{˜}{\text{Y}}$ by a single constant (*β*), that is the same for all nodes. The weight update used in NMF can then be approximated by the following learning rule applied to the node activations found (using (13) and (14)) in response to each training image:

(18) $$W⟵W⊗\left(1+\beta y\left(e^{T}-1\right)\right),$$

where *β* is a positive constant which controls the learning rate. In simulations, it was found that the weight values tend to drift upwards. This can be prevented, and learning performance improved by clipping weights at a value of one, as is done in Harpur's algorithm. The replacement of $\overset{˜}{\text{Y}}$ and the clipping of the weights means that the sequential NMF algorithm we have derived is not a particularly close approximation to the original batch NMF algorithm. However, the main purpose of this section is to make the similarity explicit between NMF and negative feedback networks and to provide a bridge to the algorithm proposed in the next section.

The sequential version of NMF described above has the same goal as the original NMF algorithm: minimising the error between the input stimulus (**x**) and the image that is reconstructed from the node outputs (**W** ^*T*^ **y**). The values of **e** indicate the degree of mismatch between the top-down reconstruction of the input and the actual input. When a value within **e** is greater than unity, indicating that a particular element of the input is underrepresented in the reconstruction, the responses of all output nodes receiving nonzero weights from the underrepresented error-detecting node are increased (via (14)) and the values of weights connecting the underrepresented error-detecting node with active output nodes are also increased (via (18)). Both these changes will lead to an increase in the strength with which that element is represented in the reconstructed image, and hence reduce the value of that element of **e** towards one (via (13)). Similarly, when a value within **e** is less than unity, indicating that a particular element of the input is overrepresented in the reconstruction, the responses of all output nodes receiving nonzero weights from the overrepresented error-detecting node are reduced (via (14)) and the values of weights connecting the overrepresented error-detecting node with active output nodes are also reduced (via (18)). Both these changes will lead to a decrease in the strength with which that element is represented in the reconstructed image, and hence increase the value of that element of **e** towards one (via (13)). When the value of **e** is equal to unity, the reconstruction of that element is perfect and the weights stop changing due to the term (**e** ^*T*^ − 1) in (18). For inputs that are not active in the input image, the corresponding elements of **e** will be zero and the corresponding weights (for active nodes) will stop changing once they have reached a value of zero.

It can be seen that when divergence-based implementation of NMF is written in terms of **e**, and converted from a batch to a sequential algorithm, that it has strong similarity to the negative feedback networks discussed in Section 2.1. Specifically, (13) is similar to (2) and (4) except that it implements a form of divisive rather than subtractive feedback. Similarly, (14) is similar to (1) and (5) except that the activation values are determined by a multiplicative rather than an additive update rule. The learning rule (18) is also similar to the rule used by the negative feedback networks (3) and (6), except that weight changes are proportional to the current value of the weight, and the value of **e** is compared to a threshold of unity. This latter difference is due to the values of **e** resulting from divisive rather than subtractive feedback: for divisive feedback perfect reconstruction of the input image results in an error value of one, whereas for subtractive feedback perfect reconstruction leads to error values of zero.

### 2.3. Divisive Input Modulation

Negative feedback networks apply subtractive inhibition to the inputs. In contrast, nonnegative matrix factorisation (in which the objective is to minimise the Kullback-Leibler divergence) can be interpreted, as shown in the previous section, as a form of divisive modulation applied to the inputs. We say “divisive modulation” of the inputs, rather than divisive inhibition, as the values of **e** generated by (13) will often be larger than the corresponding **x** value: the divisor of the division is not guaranteed to be larger than the dividend, and hence the inputs to the network could be magnified as well as inhibited.

In contrast to Fyfe's implementation of negative feedback, the divisive modulation of sequential NMF provides competition between the nodes in the network. Furthermore, unlike Harpur's implementation of negative feedback, divisive modulation is more stable (it does not lead to elements of **y** oscillating between large and small values at each iteration). We also show (in Section 3.2) that it generates better parsings of overlapping patterns. However, as has previously been observed with NMF in batch form when minimising the Kullback-Leibler divergence [8], and as will be illustrated in Section 3.3, the sequential NMF algorithm is poor at learning image components when overlap between components occurs in the training images. This section proposes improvements to the sequential NMF algorithm that results in a method for the unsupervised learning of image components that has improved performance on the tests considered in Section 3. We call this new algorithm Divisive Input Modulation (DIM).

**Figure 2:**
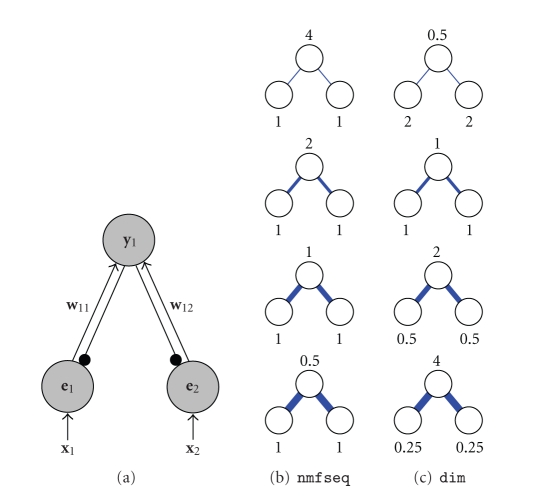
(a) A simple neural network of the type used by all the algorithms described in this article (the symbols are the same as those used in Figure 1). This network has one output node which receives equal strength weights from two error-detecting nodes (i.e., *w*_11_ = *w*_12_). Each error-detecting node receives equal strength input from two-image pixels (i.e., *x*_1_ = *x*_2_ = 1). Each subfigure in (b) and (c) shows the steady-state activation strength of the output node and the two error-detecting nodes in this simple network calculated using (b) the sequential NMF algorithm, and (c) the divisive input modulation algorithm. The steady-state responses are calculated for different weight values (indicated by the width of each connection which is proportional to its strength). From top to bottom in (b) and (c) the weights (i.e., *w*_11_ and *w*_12_) are equal to 0.25, 0.5, 1, and 2. Note that there is no stochastic element in the calculation of the neural responses generated by these algorithms, so identical results will be generated each time the network is simulated with these weight values.

Consider a single output node that receives equal strength weights from two error-detecting nodes (Figure 2(a)). When the inputs represented by the error-detecting nodes are active, then it would be expected that as the strengths of the weights increase, so would the response of the output node. However, as illustrated in Figure 2(b), the opposite happens with sequential NMF. This occurs because as the weights are increased the output node is able to more strongly inhibit the input it receives and hence the activation of the output is decreased. This keeps the reconstructed input equal to the actual input, and hence the values of **e** are equal to one. Thus, in sequential NMF, as a node becomes more strongly tuned to an input pattern its response decreases, while a node that receives only weak weights from an input pattern produces a strong response to that stimulus.

To make the variation in output response with weight strength more intuitively correct, the algorithm being proposed here calculates the response of the network as

(19) e=x⊘(ϵ+W^Ty),

(20) y⟵(ϵ+y)⊗We,

where W^=[w^1,…,w^n]T is a matrix representing the same synaptic weight values as **W** but such that the rows of W^ are normalised to have a maximum value of one. This is mathematically equivalent to calculating the residual errors using

(21) e=x⊘(ϵ+WT(y⊘w^)),

where w^ is a vector, each element of which represents the maximum synaptic weight received by the corresponding output node.

The effect of the proposed change in the calculation of the residual errors is to normalise the strength of the feedback/lateral weights, so that the maximum weight originating from each output node has a value of one. Such normalisation of inhibitory lateral weights was previously found to be advantageous for improving the competition between nodes competing to receive inputs [11, 12]. A justification for this modification, in terms of probabilities, is provided in Section 2.4. The second proposed change removes the normalisation of the output node responses, and hence makes these responses sensitive to the strength of the input weights (compare (20) with (14)). As can be seen in Figure 2(c), using the proposed method makes the response of the output node proportional to the strength of the weights. These changes also make the **e** values sensitive to the strength of the weights, and this enables the learning rule to normalise the total strength of the weights received by a node, as described below. The aforementioned changes represent a significant departure from the original NMF algorithm, and hence we have elected to give the proposed algorithm a distinct name.

The proposed learning rule is identical to that proposed for sequential NMF, that is,

(22) $$W⟵W⊗\left(1+\beta y\left(e^{T}-1\right)\right).$$

Following learning, weights are clipped at zero to ensure that they are nonnegative. As with the sequential NMF algorithm, the synaptic weights are adjusted in order to minimise the error between the input and the top-down reconstruction of the input. The learning rule increases the weights between underrepresented error-detecting nodes and active output nodes, while it decreases the weights of overrepresented error-detecting nodes and active output nodes. A weight stops changing value when the top-down reconstruction is perfect (i.e., when W^Ty=x) or when the weight is zero.

A second advantage of the proposed changes to the equations for calculating the node activation is that the values of **e** are sensitive to the scale of the weights. Hence, nodes with strong weights produce strong feedback that results in small values of **e**, whereas nodes with weak weights produce weak feedback that results in large values of **e** (see Figure 2(c)). The learning rule acts to drive the reconstruction error values towards one, which means that nodes with strong weights will have them reduced and nodes with weak weights will have them increased. Hence, learning results in the sum of the synaptic weights received by each output node being normalised to a value of one. Such normalisation is attractive from the point of view of biological plausibility, as synaptic weights cannot increase without bound. In contrast, the weights in sequential NMF are unbounded and tend to drift upwards throughout learning. As described in the previous section, clipping the weights at a value of one was found to be necessary.

### 2.4. Bayesian Interpretation of DIM

By substituting (19) into (20), the rule for updating the response of the DIM network is given by

(23) y⟵(ϵ+y)⊗W[x⊘(ϵ+W^Ty)].

If we consider a single node (*j*) and assume that this node reaches a steady-state value that is significantly greater than zero (and hence that the value of *ϵ* is insignificant), then the following condition is true:

(24) Wj[x⊘(W^Ty)]=1.

If we further assume that no other active node sends feedback to a particular input (*i*), then the relationship between this input to the network and a single active node is given by

(25) $$\frac{w_{ji}x_{i}}{{\hat{w}}_{ji}y_{j}}=1,$$

that is,

(26) $$w_{ji}=\frac{{\hat{w}}_{ji}y_{j}}{x_{i}}.$$

Bayes' theorem states that

(27) $$P(H\;|\;D)=\frac{P(D\;|\;H)P(H)}{P(D)},$$

where *H* is the hypothesis and *D* the data. If we equate *P*(*H*) (the prior) with *y*_*j*_ (the node activity), *P*(*D*) (the evidence) with *x*_*i*_ (the input activation), *P*(*H* ∣ *D*) (the posterior) with *w*_*ji*_ (the feedforward weight), and *P*(*D* ∣ *H*) (the likelihood) with ${\hat{w}}_{ji}$ (the feedback weight), then it can be seen that the relationship between the input activity and steady-state node activity is consistent with Bayes' theorem. Furthermore, the competition between nodes in the DIM network can be considered to perform “explaining away” [22]. If a node wins the competition to represent a particular input, then it inhibits other nodes from representing that input. Hence, if one hypothesis explains a particular piece of data, then support for alternative hypotheses is reduced. However, in ambiguous situations multiple hypotheses to explain a single input can each be concurrently active. The DIM network can also be considered to perform “analysis by synthesis” [23]. Hypotheses are activated by bottom-up, stimulus-driven, inputs. These hypotheses are compared to the image data and are accepted or rejected (through the competition that occurs between hypotheses) based on their ability to explain the input.

If we consider the network weights to represent conditional probabilities, then the choice to normalise the feedback weights by the maximum weight value received by each node (see previous section) makes intuitive sense. If a node represents a particular object as a conjunction of *c* inputs (lower-level feature detectors), and each of these inputs is equally weighted, then the learning rule will cause each of the feedforward weights to become equal to 1/*c*, so that the sum of the feedforward weights is equal to one. If one of these inputs is fully active, it provides 1/*c* support for the hypothesis that the object is present in the image. In the reverse direction, if the node representing the object was fully active, then the normalised feedback weights predict that each input feature should be present in the image with a probability of one. As a concrete example, consider a node that represents the category “chair.” If this node receives inputs from three-feature detectors for “seat,” “legs,” and “arms” and each of these features is weighted equally (i.e., the feedforward weights are [1/3, 1/3, 1/3]) then the presence of a seat in an image will increase the probability that the image contains a chair by 1/3. However, if the network hypothesises that the image contains a chair (with probability 1), then the probability that the image contains a seat is 1. If only half of all chairs contain arms then the feedforward weights learnt by the network would be [0.4, 0.4, 0.2], and the presence of arms in the image would provide less support for the hypothesis that the image contains a chair than the other two features. Similarly, if the network hypothesises that the image contains a chair, then the probability that the image contains a seat is still 1, but the probability that the image contains arms is half. If the feedback weights were not normalised, the hypothesis that the image contained a chair would cause the presence of a “seat” and “legs” to be predicted with a probability of 0.4, and the presence of “arms” to have a probability of 0.2. While normalising the feedback weights may not be as rigorous as learning the inverse model, it provides a better first approximation to the statistics of image formation than not normalising the feedback weights.

## 3. Results

The performance of each of the algorithms described in Section 2 were compared using a simple artificial task (described in Section 3.1). For convenience these algorithms will be referred to using the abbreviations listed in Table 1. The first set of experiments (Section 3.2) tests the ability of different algorithms to detect the component parts from which different stimuli are composed. In these experiments each network is given predefined weights and no learning occurs. These experiments thus test the ability of the activation functions to generate correct parsings of stimuli based on predefined knowledge of the possible constituents. The second set of experiments (Section 3.3) tests the ability of the different algorithms to learn the component parts from which a set of artificial training images are composed. These experiments are repeated in Section 3.4 in order to compare the performance of DIM with a wider range of algorithms, including two that have previously been shown to perform extremely well on a similar artificial task. Finally, the behaviour of the proposed algorithm on real image data is explored in Section 3.5.

### 3.1. The Squares Problem

The bars problem (and its variations) is a benchmark task for assessing the ability of algorithms to learn elementary image components [14–16, 19, 20, 24, 25]. In the standard version of this task, training images are 8 by 8 pixels in size and are made up of image components that are one-pixel wide and eight-pixel-long horizontal and vertical bars. The proportion of overlap between different image components is therefore quite small being zero for parallel bars and 1/8 for perpendicular bars. However, even with this limited degree of occlusion, nmfdiv is unable to reliably learn all the image features [8].

To test the proposed algorithm we introduce a task similar to the bars problem, but one which is more challenging due to there being more significant overlap between image components. Each artificial image was created by selecting, at random, elements from a fixed set of elementary image components. These components were all whole *s*-by-*s* pixel squares. Prior to generating an image set, each individual component was assigned a probability controlling the frequency with which it appeared in that set of images. These probabilities were selected from a uniform distribution with a range [*p*_1_, *p*_2_]. Each image was then created using the following procedure:

1. one square was chosen at random based on the probabilities assigned to each component. All other squares were independently selected to be present in the image also based on the probabilities assigned to each component. This procedure ensures that each image consists of one or more square shapes and often contains multiple, overlapping, and squares;
2. all squares present in the image were randomly assigned a “contrast” and a unique “depth.” Contrast values were randomly selected from a uniform distribution with a range [*c*_1_, *c*_2_]. Hinton and Ghahramani [25] proposed a similar variation on the bars problem in which components had randomly assigned intensities;
3. pixels in the image were given a gratscale value corresponding to the contrast of the foremost square at that pixel location, or pixels were given a greyscale value of zero if no square occurred at that location.

**Figure 3:**
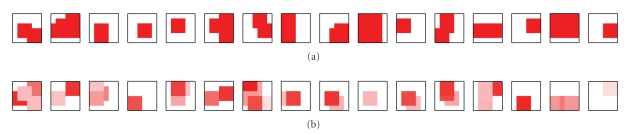
Typical examples of six-by-six pixel artificial images generated using three-by-three (i.e., *s* = 3) pixel squares components and (a) **p** = [0.1, 0.1], **c** = [1, 1], (b) **p** = [0.02, 0.2], **c** = [0.1, 1].

Typical examples of artificial images generated using this method are shown in Figure 3. The procedure described above defines a family of “squares problems.” A particular task from this set is defined by the parameters: *s* (specifying the size of the components used), **p** (defining the range of probabilities that are assigned to individual components), and **c** (defining the range of contrast values applied to squares in each image). Another possible parameter is the size of the image, but in all variations on the squares problem used in this article the image size is fixed at six-by-six pixels. Another parameter that varied between experiments was *n* the number of nodes (or equivalently basis vectors) employed by the algorithm being tested.

The degree of overlap between components is controlled by *s*. Values of two, three, and four were used for this parameter in the tasks described here. For *s* = 2 the proportion of overlap between components is either 0, 1/4, or 1/2. For *s* = 3 the proportion of overlap between components is either 0, 1/9, 2/9, 1/3, 4/9, or 2/3. For *s* = 4 the proportion of overlap between components is either 1/4, 3/8, 1/2, 9/16, or 3/4. Hence, in each case there is greater overlap between features in the squares problem than in the bars problem.

With an artificial task, like the squares problem, the underlying image components of the training and testing images are known. This allows algorithms to be quantitatively tested by comparing the components that have been represented with the known features from which the images were created. To determine which components have been represented by an algorithm, both the responses generated to test images and the weights learnt from training images can be analysed, as detailed in what follows.

#### 3.1.1. Testing Responses

The accuracy with which each algorithm could represent images was determined by analysing the responses generated by the network to test images and comparing the components represented by the active nodes with the components from which each test image was actually created. For the purposes of this analysis, any square that was selected to be in an image, but which was completely occluded by other squares, was not counted as being present in that image. Each network was tested with a set of 1000 test images. At the very minimum, in order to be able to distinguish those components that are present in an image from those that are absent it is necessary for nodes that represent the components making up an image to have greater activity than all other nodes (and for nodes representing components not present in the image to generate a weaker response than all the nodes representing active components). More formally, if ${\overset{ˇ}{y}}_{t}$ is the minimum activity across all the nodes representing squares in the image, and ${\hat{y}}_{f}$ is the maximum activity across all the nodes representing squares not in the image, then for a correct representation of that image we require $y>{\hat{y}}_{f}$ for all nodes representing image components present in the image, and $y\leq {\overset{ˇ}{y}}_{t}$ for all nodes representing image components absent from the stimulus. It follows that the number of responses which are false negatives is given by the number of nodes which represent a components present in the image but which are not active (i.e., for which $y\leq {\hat{y}}_{f}$). The number of responses which are false positives is given by the number of nodes that represent components not in the image but which are active (i.e., for which $y>{\overset{ˇ}{y}}_{t}$).

In tests on image parsing (Section 3.2) the networks were hard wired to represent each image component. Hence, it was known which nodes represent which image components and this information was used to perform the above analysis. For tests in which image components were learnt (Sections 3.3 and 3.4), it was necessary to determine which node represented which components (in order to determine which nodes should and should not be active in response to the components known to make up each test image). Hence, prior to carrying out the above analysis the selectivities of each node were calculated. Each node was assumed to represent that component for which it had the highest selectivity, with the additional constraint that each component was allocated to a distinct node. Selectivity was measured as the difference in the mean response of a node to all the test images that contained a component and the mean response to all the other test patterns (which did not contain that component). Since allocating a single node to represent multiple components would automatically result in responses unable to distinguish patterns, we also required that each component was allocated to a distinct node. Hence, if the above procedure resulted in more than one component being allocated to a single node, the component for which the node had the highest selectivity was allocated to that node, and the nodes with the next highest selectivities were allocated to representing the remaining components. This process was repeated until all components were allocated to distinct nodes.

#### 3.1.2. Testing Weights

The accuracy with which each algorithm could learn weights selective to image components was tested by comparing the weights formed following training with the image features from which the training images were created. A node was considered to represented an image component if the following criteria were met: (1) the sum of the synaptic weights corresponding to that image component was at least three times greater than the sum of all other weights received by the node; (2) each individual weight corresponding to the image component was greater than any weight received by the node from an input not forming part of the component; (3) each individual weight corresponding to the image component was greater than the mean of all the weights received by the node. The first criteria ensures that a node is strongly selective for a particular individual component. The second criteria ensures that the node does not represent pixels that do not form part of the component. The third criteria ensures that a node represents all pixels that form part of that component. By applying these criteria for all image components to each node, the number of features represented by distinct nodes in the network was determined.

Note that testing weights is complementary to testing responses, and these two tests may give very different results. For example, it may be possible for nodes to represent only certain pixels that form each component, but to still reliably respond to the presence of each component. In this case the response analysis might yield a low error rate while the weight analysis would suggest that few components were represented. On the other hand, it might be possible for multiple nodes to learn very similar weights representing all the pixels of a single component. The competition between these nodes might cause the same component to be represented by a different node in different contexts. In this case the response analysis would suggest poor performance, while the weight analysis would indicate the component was accurately represented. Hence, both a low parsing error rate and a high percentage of components represented by the weights are required to indicate the success of a learning algorithm.

### 3.2. Parsing Images into Elementary Components

An image usually consists of a number of different objects, parts, or features and these components can occur in different configurations to form many distinct images. Identifying the underlying components which are combined to form an image is thus essential for generating an accurate representation of a visual scene and is necessary for performing object recognition. If the underlying image components are known and a neural network is given predefined weights so that all possible components are represented by distinct nodes, then the ability of the network to parse an image can be assessed (as described in Section 3.1.1) by comparing the activity generated in response to a test image with that expected to be generated in response to the components known to be present in the test image.

**Figure 4:**
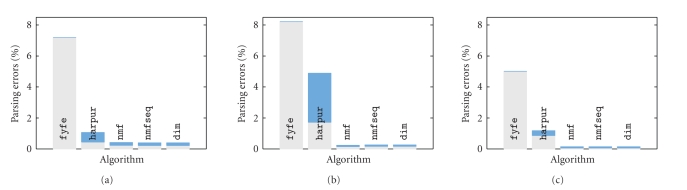
Errors in parsing the overlapping squares tasks with (a) *s* = 2, (b) *s* = 3, and (c) *s* = 4; **p** = [0.1, 0.1], and **c** = [1, 1] in each case. Each bar shows the proportion of errors generated across 1000 test images. Each bar is subdivided into the proportion of false negatives (lighter, lower, section) and the proportion of false positives (darker, upper, section).

Figure 4 shows the percentage of false negatives and false positives generated by each algorithm for experiments using two-by-two, three-by-three, and four-by-four pixel square components. In each case, all components appeared in the test images with equal frequency (**p** = [0.1, 0.1]) and all components were presented in every image with the same contrast (**c** = [1, 1]). For each test the number of nodes in the network was made equal to the number of image components in each task. It can be seen that algorithms dim, nmfseq, and nmfdiv all produce accurate parsings of the images with only a very small proportion of errors in the representations generated. Across all three tests dim, nmfseq, and nmfdiv generate parsings that are at least 99.6% correct. The results are very similar, due to the similar mechanism of divisive input modulation used in these three algorithms. In comparison, algorithm harpur produces poorer results across all three tasks with total errors of up to 4.9%. The lack of competition in algorithm fyfe results in this algorithm producing the worst performance in all the tasks, with up to 8.2% errors. Note that all the errors for fyfe are false negatives, this is due to nodes representing components not present in the image being allowed (due to the lack of competition) to generate a response equal in strength to the smallest response generated to a component that is present.

**Figure 5:**
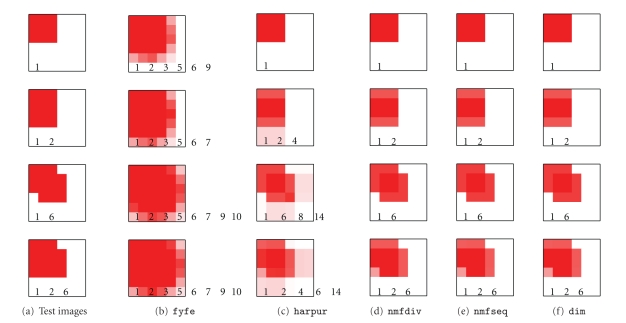
Node activations generated in response to input images containing overlapping 3 × 3 pixel square components. (a) Test images. Numbers indicate which components are present in each test image. (b)–(f) Images reconstructed from the response of each network. Numbers indicate which components are represented by strongly active nodes in each network (nodes that have an output activation greater than the mean of all node activations). Each network was given predefined weights so that distinct nodes represented 3 × 3 pixel squares at all possible locations.

Figure 5 illustrates parsings produced by each algorithm for the three-by-three squares task. Algorithms dim, nmfseq, and nmfdiv correctly represent the components present (and only those components) in each test image. In contrast, both algorithms harpur and fyfe represent more components than are actually present in the images. For Fyfe's algorithm this is to be expected, as there is no competition between the nodes. For Harpur's algorithm it demonstrates that the competition is not particularly successful in determining which nodes represent components that are actually present in the image.

### 3.3. Learning Elementary Image Components

The previous section explored the ability of different mechanisms of competition to identify the elementary features from which an artificial image was composed. Networks were given predefined synaptic weights, and hence, knowledge of the component features was built into each network. In this section, the ability of each algorithm to learn elementary image components is tested. Each network is trained using a randomly generated sequence of artificial images each of which is generated from a predefined set of image components. Training images were created using the procedure described in Section 3.1. Hence, images contained multiple overlapping square components. Nine versions of the squares task were used in total, with three variations of the task being performed for each of the three different component sizes used (*s* = 2, *s* = 3, and *s* = 4). In the first variation, all the components had the same probability of occurring in the training images (**p** = [0.1, 0.1]), each component had an equal contrast (**c** = [1, 1]), and each network had exactly the same number of nodes as there were components in the training data. In the second variation, the training data was generated identically to the first variation, but networks contained a fixed, excess, number of nodes. Forty-eight nodes were used, this was an arbitrary figure chosen to ensure that there was a large excess of nodes in all experiments. In the third variation of the task, each component had a different probability of occurring in the training images (**p** = [0.02, 0.2]), in each image each component was randomly assigned a contrast (**c** = [0.1, 1]), and 48 nodes were used.

Each algorithm was given random initial weight values and was trained using a set of 1000 training images. Ten trials were performed for each combination of algorithm and task. Each trial used a different, randomly selected, set of training images and different, randomly generated, initial weight values. Parameter values that gave the best results were found by trial and error, and were kept constant across variations in the task (see Table 2). Parameter values were fixed to assess the ability of each algorithm to robustly learn image components across a number of variations in the task. In order to succeed at all the variations in the squares task tested here, an algorithm needed to cope with changes to the size of components, the frequency of appearance of components, the greyscale values of components, and the number of nodes used to represent those components (i.e., to changes in parameters *s*, **p**, **c**, and *n*).

**Figure 6:**
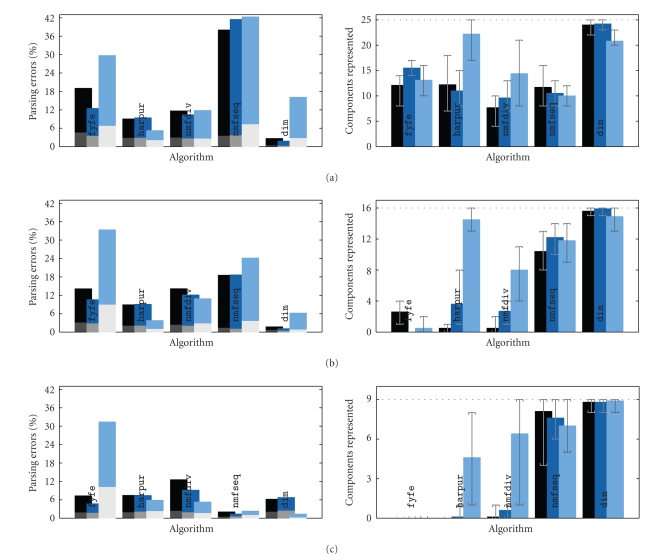
Performance of each algorithm when trained on the overlapping squares task with (a) *s* = 2, (b) *s* = 3, and (c) *s* = 4. Results are shown for three different versions of each task; foreground bars show results when *n* equals the number of image components, **p** = [0.1, 0.1], and **c** = [1, 1]; middle bars show results for *n* = 48, **p** = [0.1, 0.1], and **c** = [1, 1]; background bars show results for *n* = 48, **p** = [0.02, 0.2], and **c** = [0.1, 1]. Results are averaged over 10 trials for each condition. Plots in the left-hand column show the mean number of errors generated in the response of each network to 1000 test images. Each bar is subdivided into the proportion of false negatives (lighter, lower, section) and the proportion of false positives (darker, upper, section). Plots in the right-hand column show the mean number of components correctly represented by the synaptic weights learnt by each algorithm. Error bars show best and worst performance, across the 10 trials.

Following training the synaptic weights were fixed and the response of each network to 1000 randomly generated test images was recorded. Test patterns were generated using the same procedure as used to generate the training images with **p** = [0.1, 0.1] and **c** = [1, 1]. The proportion of responses that were false negatives and false positives were determined using the procedure described in Section 3.1.1. These results, averaged over ten trials, are shown in the left column of Figure 6. In addition to testing how well each algorithm could parse images following training, the weights learnt by each algorithm were also assessed as described in Section 3.1.2. The average number of components successfully represented by the weight values learnt by each algorithm are shown in the right column of Figure 6.

It can be seen that across all the variations in the task, algorithm dim produced the best overall results; the weights learnt corresponded to the image components and the subsequent parsing errors were small. The performance of dim was unaffected by changing parameters *s*, **p**, **c**, or *n*, although excess nodes generally improved performance slightly. Similarly, the results produced by algorithms fyfe, harpur, nmfdiv, and nmfseq were unaffected by variations in parameters **p** and **c**, suggesting that none of the algorithms tested had a strong prior expectation (explicit or implicit to the algorithm) for the frequency of appearance, or the greyscale, of image components. However, in contrast to dim, the number of nodes in the network had a large effect on the results produced by algorithms harpur and nmfdiv. When an excess of nodes was used, these algorithms produced very poor results, suggesting that these algorithms require prior knowledge of the number of components. In general, it is not known in advance how many components need to be represented. Hence, a practical algorithm needs to be able to correctly learn image components with an excess of nodes. Furthermore, in contrast to dim, the performance of algorithms fyfe, harpur, nmfdiv, and nmfseq was affected by the size of the image components. The analysis of the weights shows that the performance of algorithm nmfseq improved as *s* increased, whereas, the performance of fyfe, harpur, and nmfdiv deteriorated as *s* increased (i.e., as the overlap between image components became larger). As noted in Section 2.2.1 following (18), the learning rule implemented in the sequential NMF algorithm is not a very close approximation to that of the original batch NMF algorithm. Hence, while the results for parsing are very similar, the learning results for nmfdiv and nmfseq differ significantly.

### 3.4. Benchmarking

The previous sections have compared the performance of the proposed algorithm with those previous algorithms from which it derives. It has been shown that dim is more successful than its predecessors in learning the squares problem. However, to be of general interest it is necessary to show that the performance of dim is competitive with other algorithms which are known to be able to learn overlapping image components. In this section the experiments reported in Section 3.3 are repeated with a number of alternative algorithms; enhanced versions of the three algorithms from which dim originates, and algorithms that have previously been shown to produce state-of-the-art performance on a similar set of tasks (i.e., the bars problem).

Charles et al. [15] presents a modified version of algorithm fyfe, which it is claimed produces better performance on the bars problem (although no quantitative results are provided). It is also claimed that this modified algorithm can learn to represent individual bars even when the training images contain many coactive bars (e.g., using images that contain seven coactive bars). However, in this case the training images were created using the additional constraint that all bars had the same orientation. Hence, there is zero overlap between the components in this data. This modified algorithm (which we will call fyfe2) replaces (1) with

(28) $$y={\left\{Wx\right\}}^{+}+n,$$

where {·}^+^ denotes that the positive half-wave rectified value of the node activations are taken, and **n** is a vector of noise values added to these nonnegative node activations. These noise values are taken from a Gaussian distribution with zero mean and a standard deviation of *ν*. Also, in contrast to algorithm fyfe, the weights are allowed to take both positive and negative values.

Harpur [17] proposes a large range of possible variations on algorithm harpur. These differ in the constraints placed on the weights, constraints placed on the node activations and the learning rule employed. One variation that is particularly successful in learning the squares task is an alternative learning rule proposed in Harpur and Prager [18]. This replaces (6) with

(29) $$W⟵W+\beta y\Theta (x^{T}),$$

where Θ(**x** ^*T*^) performs a thresholding function on the elements of **x** ^*T*^ (i.e., Θ(**x** ^*T*^) = [*x*_1_ − *θ*,…, *x*_*m*_ − *θ*]).

A large number of variations on NMF have been proposed [1–7, 9, 26, 27]. The nmfsc algorithm [3] has previously been found to produce good performance on the bars problem, even for modified versions of this task where the overlap between bars is increased [8]. The nmfsc algorithm allows optional constraints to be imposed on the sparseness of either the basis vectors, the activations, or both. The constraint on the sparseness of the basis vectors (*s*_*W*_) can range from 0 (which would produce completely distributed basis vectors) to a value of 1 (which would produce completely sparse basis vectors). This parameter affects the sparseness of the rows of **W** to ensure that each node learns to represent a component with a similar prespecified fraction of active pixels. The constraint on the sparseness of the activations (*s*_*Y*_) is also in the range [0, 1] and is applied to the rows of **Y**. Hence, ensuring that each component is present in a similar prespecified fraction of the training images.

The preintegration lateral inhibition or dendritic inhibition algorithm (di) has been shown to outperform a wide range of other algorithms on the bars problem [8, 12, 28]. The similarity of the squares task to the bars task suggests that the dendritic inhibition algorithm will provide a good benchmark for assessing the proposed algorithm. Algorithm di also has strong similarity to the other algorithms discussed in this article, as it employs a mechanisms of competition through which nodes within a population suppress the inputs to neighbouring nodes. It may therefore also be considered to be a predecessor of dim.

**Figure 7:**
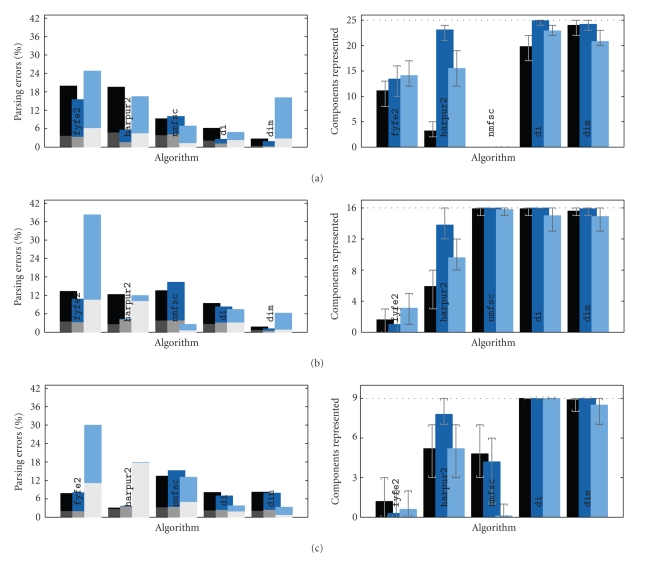
Performance of the benchmarking algorithms when trained on the overlapping squares task with (a) *s* = 2, (b) *s* = 3, and (c) *s* = 4. See caption of Figure 6 for details. Note that the results for algorithm dim are identical to those shown in Figure 6 but are reproduced here for ease of comparison.

The parameter values, that gave the best results for these additional algorithms were found by trial and error, and were kept constant across all the variations in the squares task. These values are listed in Table 3. Results for experiments identical to those described in Section 3.3 are shown in Figure 7. It can be seen that dim produced results that are very comparable to those of the di algorithm and that these results were far superior to those of the other algorithms tested. Algorithm fyfe2 produced results that were only marginally better than fyfe, with performance still deteriorating with increasing overlap. Algorithm harpur2 produced results that were significantly better than harpur. One improvement was that the algorithm performed well when trained using excess nodes. However, performance was poor on the version of the squares problem in which components had an unequal probability of appearance and varying contrast. This is likely to be due to the fixed threshold applied to **x** in the modified learning rule. The performance of the nmfsc algorithm depended critically on the particular sparseness parameters that were chosen, particularly parameter *s*_*W*_ which determines the sparseness of the weights learnt by each node. With *s*_*W*_ = 0.5 this algorithm produced excellent performance learning intermediate sized squares (i.e., for *s* = 3) but poor performance for *s* = 2 and *s* = 4. Good results for squares tasks with other size components could be achieved by varying parameter *s*_*W*_ but no single parameter value could produce good results across all the values of *s* used in these experiments. Hence, while an appropriate choice of sparseness constraint improves performance over algorithm nmfdiv, an inappropriate choice prevents the identification of factors that have a different size to that specified by the sparseness parameter chosen. Furthermore, when components have a variety of sizes (as was the case in these experiments) no sparseness constraint exists that will allow all those components to be successfully learnt.

### 3.5. Image Data

**Figure 8:**
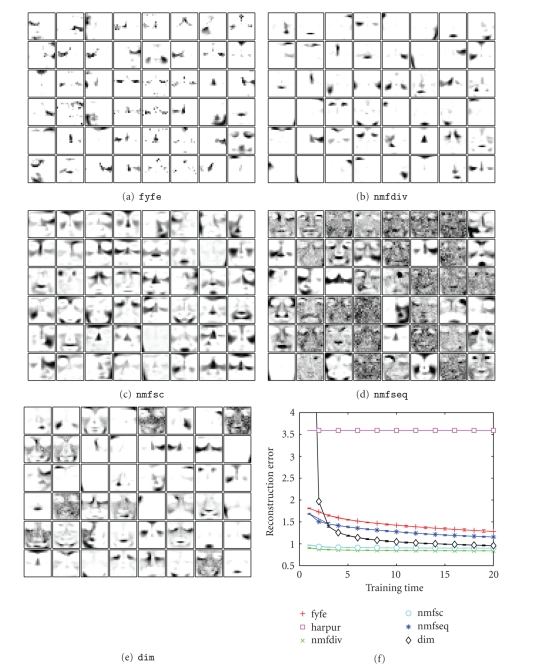
(a)–(e) Example basis vectors learnt by each algorithm when trained on the CBCL Face Database, with *n* = 48. (f) The change during the course of training of the mean Euclidean distance between the input and the reconstructed input. Results show mean of 10 trials performed using each algorithm. The best and worst performance over these 10 trials is shown by the error bars (which are very small in each case). Note that the total training time used for each algorithm (and hence the meaning of the value “20” on the *x*-axis of this graph) was 200 epochs for fyfe, 2000 epochs for algorithms nmfdiv and nmfsc, and 20 epochs for all the other algorithms. Training times are therefore not all directly comparable: at any particular time fyfe has seen 10 times more data and been updated 10 times more than dim, while nmfdiv and nmfsc have seen 100 times more data but been updated 24 times less than dim.

It has become common practise to test NMF algorithms using the CBCL Face Database. (CBCL Face Database *#*1, MIT Center For Biological and Computation Learning, http://cbcl.mit.edu/software-datasets/FaceData2.html.) The weight vectors learnt by algorithm dim when applied to this task are shown in Figure 8(e). Parameter values were identical to those used to learn the squares problem. The preprocessing of the images was identical to that performed in [4]. It can be seen that the proposed algorithm learns components that are holistic, partially localised (e.g., right and left halves of a face, cheeks plus nose, etc.), and localised (chin, lips, eyebrows, etc.). Algorithm fyfe also learns components that are localised and partially localised (Figure 8(a)). In contrast, nmfdiv learns basis vectors that correspond to localised image parts (Figure 8(b)) as found in previous work [1, 3, 4]. While algorithms nmfsc (using the same sparseness constraints used to learn the squares task) and nmfseq learn both holistic and semilocalised image components (see Figures 8(c) and 8(d)). Algorithm harpur fails to learn any components due to all node activations oscillating between large and small values at each iteration. Due to this unstable behaviour all node activations are zero at the end of the 100 iterations performed to find the steady-state node activations in response to each training pattern, and hence, the weights never change from the original random ones.

For this test case, what constitutes a meaningful representation is unknown. However, we can gauge how accurately each algorithm represents the training data by calculating the Euclidean distance between the input image and the input reconstructed from the node activations. Figure 8(f) shows the mean reconstruction error, averaged over all 2429 training images, at various times during the training of each algorithm. Identical parameters to those used for learning the squares data have been used in each case, and each algorithm has been trained with 48 nodes or basis vectors. The only difference was that the training times for the online learning algorithms were increased by a factor of 2.429 due to the training set containing 2429 images rather than the 1000 images used previously. This keeps the relative number of training epochs for each algorithm constant. It can be seen that the reconstruction error for dim is initially the highest. This is due to dim being sensitive to the scale of the weights. However after training, dim is able to reconstruct the images with an accuracy approaching the batch NMF algorithms. Note that harpur fails to learn due to all node activations oscillating between zero and large values at each iteration, and that the reconstruction error for nmfdiv and nmfsc is flat as the weights learnt by these algorithms have already converged to their final values by 200 epochs (corresponding to a training time of 2 in Figure 8(f)).

## 4. Discussion

Fyfe's negative feedback algorithm employs subtractive inhibition of the network inputs in order to affect learning. The feedback inhibition does not directly affect output activations, but only has an indirect effect through subsequent synaptic weight changes. There is thus no direct competition between nodes for the right to be active in response to a stimulus. It has been shown that various versions of this algorithm are capable of learning weights that represent individual image components in tasks (such as the bars problem) where image components have only a small overlap [13–16]. When there is little overlap between the representations learnt by each node, it is also possible for the activation of the network to show distinct responses for different stimuli. However, when image components have strong overlap, the lack of competition between the nodes means that the network fails to accurately represent the input it receives even if nodes have correctly learnt weights that are selective to patterns within the stimulus. This is illustrated in Section 3.2 where it is shown that input patterns generate responses from many nodes other than those that represent the stimuli forming the input, even when the synaptic weights have been hard wired to provide a perfect representation of each individual image component. Hence, the lack of competition in Fyfe's algorithm results in the network being unable to express the knowledge that has been encoded in its synaptic weights. Unsurprisingly, Fyfe's algorithm is also poor at learning image components that overlap (see Sections 3.3 and 3.4). This is also likely to be due to the nonspecific responses generated to input patterns being fed into the activity-dependent learning rule.

The failure of Fyfe's algorithm to provide competition between nodes is rectified in Harpur's negative feedback network. This is achieved by allowing the inhibited inputs to affect output responses. This provides a mechanism for competition between the nodes in the network which enables the components forming an image to be identified; there is selective activation of those nodes representing stimuli present in the input. However, the results in Section 3.2 show that the competition is not always sufficiently selective, enabling nodes that represent stimuli not present in the input to be active. Furthermore, by using subtractive inhibition Harpur's algorithm can become unstable. Subtraction can result in the inhibited inputs all becoming zero or negative, this can in turn lead to all outputs becoming zero. This will then lead to no inhibition being applied to the inputs at the next iteration and subsequently output activations becoming large. Oscillations can therefore occur during the iterative process used to determine the network activation. Such instability resulted in Harpur's algorithm failing to learn image components of real images in Section 3.5.

Using divisive, rather than subtractive, feedback can avoid instability: division can only result in inputs becoming small, rather than disappearing entirely. In addition, the results in Section 3.2 show that divisive input modulation (as employed in NMF and DIM) results in more selective parsings. Competition in nmfdiv, nmfseq, and dim is mathematically very similar, and hence these algorithms produce almost identical results when hard wired with identical synaptic weights. However, these algorithms produce very different results when applied to learning image components.

The learning process in NMF attempts to minimise the error between the input to the network and the input that is reconstructed from the outputs of the network. Minimising the reconstruction error causes nodes to learn parts of input patterns that are not already represented by other nodes. The result is that nodes tend to learn nonoverlapping portions of the elementary image components rather than whole components. These random pieces of stimuli are not meaningful representations of the image data. In order to learn the features from which the training images are composed, NMF would require either that each component occupied a distinct spatial location or that the superposition of image components resulted in a linear combination of sources [8]. However, in reality objects or object parts do not occupy unique and distinct locations nor does the superposition of objects or object parts usually result in a linear combination of sources but, due to occlusion, generally results in a nonlinear combination. It is possible to overcome the problem caused by overlap by imposing additional constraints on the objective function, as in the nmfsc algorithm [3]. However, these constraints themselves prevent NMF from identifying image components that violate the imposed constraint (see Section 3.4).

**Figure 9:**
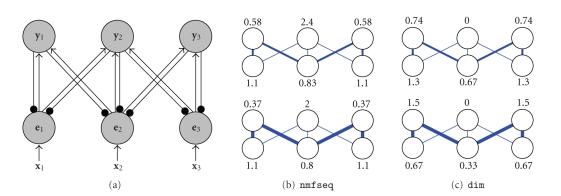
(a) A simple neural network of the type used by all the algorithms described in this article (the symbols are the same as those used in Figure 1). This network has three output nodes which receive input from three error-detecting nodes. All three error-detecting nodes receive equal strength input from three image pixels (i.e., *x*_1_ = *x*_2_ = *x*_3_ = 1). The first output node has weights that are selective to the first two inputs (i.e., *w*_11_ = *w*_12_ = *a*, where *a* > 0 while *w*_13_ = 0 and is thus missing from the diagram), and the third output node represents the last two inputs (i.e., *w*_31_ = 0 while *w*_32_ = *w*_33_ = *a*, where *a* > 0). The middle output node has weak weights (equal to 0.25) connecting it to all three error-detecting nodes. Each subfigure in (b) and (c) shows the steady-state activation strength of the three output nodes and the three error-detecting nodes in this simple network calculated using (b) the sequential NMF algorithm, and (c) the divisive input modulation algorithm. The steady-state responses are calculated for different values of *a* (the positive weights targeting the first and third output nodes). In the top row of (b) and (c) *a* equals 0.5, and in the bottom row of (b) and (c) *a* equals 1 (the width of each connection in these subplots is proportional to its strength). Note that there is no stochastic element in the calculation of the neural responses generated by these algorithms, so identical results will be generated each time the network is simulated with these weight values.

The proposed algorithm (DIM) also attempts to minimise the error between the input image and the image reconstructed from the node outputs. However, it succeeds in learning complete image features rather than random parts of image features. The principal reason for this is the change in the activation function. As described in Section 2.3, and illustrated in Figure 2(b), NMF employs an activation function that behaves counterintuitively. As a node becomes more strongly tuned to a particular input pattern, its response decreases rather than increases. Another problem with this activation function is illustrated in Figure 9(b). Two output nodes representing partially overlapping input features (pixels 1 and 2, and pixels 2 and 3) both respond when these features are present in the image simultaneously. However, a third node that is not strongly tuned to either feature will be even more active, and hence will adjust its weights to learn the nonoverlapping parts of both input components (i.e., the inputs for which the values of **e** are greater than one). This problem is not solved by the nodes learning to become better representations of the image features, since as the weights increase, the response decreases and hence the untuned node is even more strongly activated relative to the tuned nodes. As illustrated in Figure 9(c), this problem does not occur with the activation rule proposed in Section 2.3. Here, the nodes that strongly represent the image features are the ones that become active and these nodes succeed in suppressing the activation of the untuned node.

In tasks where there is significant overlap between image components, and excess nodes in the network (i.e., the four-by-four squares problem with *n* = 48), there is still a tendency for DIM to learn parts of image components rather than whole components. Initially, representations are learnt correctly, but multiple nodes represent the same component. After further training these competing nodes divide the components into multiple subparts. Various methods have been found to improve stability: increasing the number of iterations performed by the algorithm; gradually reducing the learning rate (i.e., the value of the *β*) throughout training; modifying the learning rule to cause weights to gradually decay towards zero unless this effect is counteracted by a node being active sufficiently frequently to increase its weights; or not reinitialising the values of **y** when each new training image is presented to the network. However, none of the these techniques have been used to generate the results presented here, and this issue of stability is far less severe with the proposed algorithm than with the other algorithms tested. In general, the proposed algorithm accurately learns to represent the elementary components from which images are composed, even when those components have considerable overlap. Furthermore, DIM is capable of accurately representing the components present in an image following learning. Hence, the divisive input modulation algorithm performs extremely well on the tasks reported here. Indeed its performance on the squares task is comparable to the dendritic inhibition algorithm that has previously been shown to outperform a wide range of other methods on the bars problem [8, 12, 28]. However, the computational complexity of the dendritic inhibition algorithm is far in access of DIM.

By employing an online, rather than a batch, learning procedure DIM has greater biological plausibility than NMF. The proposed activation rules have a similar level of biological plausibility to the other algorithms discussed. Divisive input modulation could either be implemented in cortical circuits via divisive feedback inhibition originating in one cortical layer and targeting the outputs of neurons in the preceding cortical layer, or via divisive lateral inhibition targeting the dendrites of neurons within the same cortical layer [11]. The proposed learning rule is also biologically plausible as it employs only information that is local to each synapse (the pre- and postsynaptic activity and the current synaptic weight value). It also has the added advantage of automatically normalising the sum of the weights received by each node. Hence, synaptic weights cannot increase without bound and no separate normalisation procedure is required as is the case with many other algorithms.

## 5. Conclusion

It has been shown that nonnegative matrix factorisation and negative feedback neural networks are mathematically similar. Both NMF and negative feedback networks attempt to adjust synaptic weights and neural responses in order to minimise the error between the input stimulus and the input that is reconstructed from the node outputs. However, the mechanism for calculating this reconstruction error differs with NMF using a divisive mechanism and negative feedback networks using a subtractive mechanism. By recognising the correspondence between these existing algorithms we have derived a new neural network algorithm that combines aspects of both NMF and negative feedback networks. The new algorithm is similar to Harpur's negative feedback network in that it performs online learning and uses an iterative procedure to calculate the neural activations. It is also similar to NMF (implemented using the Kullback-Leibler divergence as the objective function) in that it employs an equivalent learning rule and uses a divisive rather than subtractive form of feedback. However, the proposed algorithm improves upon both these existing methods by being capable of successfully learning meaningful elementary image components even in the presence of considerable occlusion, and on the tasks considered here, it significantly outperforms a number of existing methods when applied to learning overlapping image components. The proposed algorithm can be interpreted as a neural implementation of Bayesian inference and combines mathematical simplicity and biological plausibility with reliable learning and recognition characteristics.

## Figures and Tables

**Table 1:** Summary of the algorithms tested in this article.

Acronym	Description	Definition
fyfe	Fyfe's algorithm for negative feedback	Equations (1), (2), and (3)
harpur	Harpur's algorithm for negative feedback	Equations (4), (5), and (6)
nmfdiv	Nonnegative matrix factorisation with divergence objective	Equations (8) and (9)
nmfseq	Sequential nonnegative matrix factorisation	Equations (13), (14), and (18)
dim	Divisive input modulation	Equations (19), (20), and (22)

**Table 2:** Details of the training procedure used for each of the algorithms tested. In all cases the parameter values listed were those found to produce the best results. Parameter values were kept constant across variations in the task. All algorithms except nmfdiv use an online learning procedure. Hence, each weight update occurs after an individual training image has been processed. This is described as a training cycle. In contrast, nmfdiv uses a batch learning method. Hence, each weight update is influenced by all training images. This is described as a training epoch. Hence, with a set of 1000 training images (as used in these experiments) an epoch is equivalent to 1000 training cycles for the online learning algorithms. The third column specifies the number of iterations used to determine the steady-state activations values. Weights were initialised using random values selected from a Gaussian distribution with the mean and standard deviation indicated. In each case initial weights with values less than zero were made equal to zero.

Algorithm	Training time	Iterations	Weight initialisation	Parameter values
fyfe	200 000 cycles	n/a	mean = $\frac{1}{8}$, std = $\frac{1}{32}$	*β* = 0.0001
harpur	20 000 cycles	100	mean = $\frac{1}{8}$, std = $\frac{1}{32}$	*β* = 0.1, *μ* = 0.025
nmfdiv	2 000 epochs	n/a	mean = $\frac{1}{2}$, std = $\frac{1}{8}$	n/a
nmfseq	20 000 cycles	50	mean = $\frac{1}{4}$, std = $\frac{1}{16}$	*β* = 0.05
dim	20 000 cycles	50	mean = $\frac{1}{16}$, std = $\frac{1}{64}$	*β* = 0.05

**Table 3:** Details of the training procedure used for each of the benchmarking algorithms tested. In all cases the parameters values listed were those found to produce the best results. Parameter values were kept constant across variations in the task. All algorithms except nmfsc use an online learning procedure and hence training time is measured in cycles, whereas for nmfsc training time is measured in epochs. See the caption of Table 2 for further details.

Algorithm	Training time	Iterations	Weight initialisation	Parameter values
fyfe2	200 000 cycles	n/a	mean = $\frac{1}{4}$, std = $\frac{1}{32}$	*β* = 0.01, *ν* = 0.1
harpur2	20 000 cycles	50	mean = $\frac{1}{32}$, std = $\frac{1}{8}$	*β* = 0.0025, *μ* = 0.05, *θ* = 0.5
nmfsc	2 000 epochs	n/a	mean = $\frac{1}{2}$, std = $\frac{1}{8}$	*s*_*W*_ = 0.5, *s*_*Y*_ = none
di	20 000 cycles	25	mean = $\frac{1}{36}$, std = 0.001	*β* ^+^ = 0.25, *β* ^−^ = 0.25
dim	20 000 cycles	50	mean = $\frac{1}{16}$, std = $\frac{1}{64}$	*β* = 0.05
